# Improving child survival through a district management strengthening and community empowerment intervention: early implementation experiences from Uganda

**DOI:** 10.1186/s12889-015-2129-z

**Published:** 2015-08-19

**Authors:** Anne Ruhweza Katahoire, Dorcus Kiwanuka Henriksson, Eric Ssegujja, Peter Waiswa, Florence Ayebare, Danstan Bagenda, Anthony K. Mbonye, Stefan Swartling Peterson

**Affiliations:** Child Health and Development Centre, Makerere University, Kampala, Uganda; Karolinska Institutet, Solna, Sweden; Uppsala University, Uppsala, Sweden; School of Public Health, Makerere University College of Health Sciences, Kampala, Uganda; College of Public Health, University of Nebraska Medical Center, Omaha, USA; Harvard T.H. Chan School of Public Health, Harvard University, Boston, USA; Ministry of Health, Kampala, Uganda

## Abstract

**Background:**

The Community and District Empowerment for Scale-up (CODES) project pioneered the implementation of a comprehensive district management and community empowerment intervention in five districts in Uganda. In order to improve effective coverage and quality of child survival interventions CODES combines UNICEF tools designed to systematize priority setting, allocation of resources and problem solving with Community dialogues based on Citizen Report Cards and U-Reports used to engage and empower communities in monitoring health service provision and to demand for quality services. This paper presents early implementation experiences in five pilot districts and lessons learnt during the first 2 years of implementation.

**Methods:**

This qualitative study was comprised of 38 in-depth interviews with members of the District Health Teams (DHTs) and two implementing partners. These were supplemented by observations during implementation and documents review. Thematic analysis was used to distill early implementation experiences and lessons learnt from the process.

**Results:**

All five districts health teams with support from the implementing partners were able to adopt the UNICEF tools and to develop district health operational work plans that were evidence-based. Members of the DHTs described the approach introduced by the CODES project as a more systematic planning process and very much appreciated it. Districts were also able to implement some of the priority activities included in their work plans but limited financial resources and fiscal decision space constrained the implementation of some activities that were prioritized. Community dialogues based on Citizen Report Cards (CRC) increased community awareness of available health care services, their utilization and led to discussions on service delivery, barriers to service utilization and processes for improvement. Community dialogues were also instrumental in bringing together service users, providers and leaders to discuss problems and find solutions. The dialogues however are more likely to be sustainable if embedded in existing community structures and conducted by district based facilitators. U report as a community feedback mechanism registered a low response rate.

**Conclusion:**

The UNICEF tools were adopted at district level and generally well perceived by the DHTs. The limited resources and fiscal decision space however can hinder implementation of prioritized activities. Community dialogues based on CRCs can bring service providers and the community together but need to be embedded in existing community structures for sustainability.

**Electronic supplementary material:**

The online version of this article (doi:10.1186/s12889-015-2129-z) contains supplementary material, which is available to authorized users.

## Background

In 2013, an estimated 6 · 3 million children worldwide died before the age of 5 years [1]. Infectious causes accounted for 51.8 % of all death with the leading causes being Pneumonia (14 · 9 %), diarrhea (9 · 2 %), and malaria (7 · 3 %) [2]. In Africa, Pneumonia and Diarrhea accounted for 17 and 12 % of deaths respectively [2]. As a response to both the importance of these two diseases and lack of progress over the past decade, UNICEF and the World Health Organization (WHO) issued two reports, the Global Action Plan for the Prevention and Control of Pneumonia (2007) [3] and “Diarrhea: Why Children Are Still Dying and What Can Be Done,” (2009) [4]. Both call for the implementation of a package of interventions across the promote-prevent-and treat continuum. If these pneumonia and diarrhea interventions were to achieve universal coverage, cause-specific mortality would be reduced in African settings by an estimated 70 % for pneumonia and by over 90 % for the diarrhea [5].

While selective interventions can make a major difference, these still have a low coverage [6, 7]. The reasons for poor implementation are several-fold, as outlined in an evaluation of a large project designed to reduce deaths from the main killers of children [8]. These include failure to prioritize those interventions that are most likely to prevent deaths, problems with the supply and management of essential commodities such as vaccines, ORS, and antibiotics, and the absence of community-based care. This situation creates a major challenge to implementation at district level, and substantial reduction in deaths from pneumonia and diarrhea will not be possible unless district administration of commodities and community-based service delivery are improved and are focused upon achieving tangible results. In many African countries, these problems have been compounded by an increasing tendency to decentralize services that has not been accompanied by adequate strengthening of district management and local performance assessment [8, 9]. An additional problem affecting implementation is that the communities are often passive players and are rarely consulted.

Almost two-thirds of all child deaths could be prevented through available interventions [10] that have been proven to be effective and affordable [11–13]. Delivery of these interventions requires broad strengthening of health systems since many factors may influence how services are delivered, for example: information systems, governance, human resources and more traditional concerns of knowledge, skills, and availability of technology [11–13].

One of the obstacles is failure to adequately implement and deliver these evidence-based essential interventions that could prevent death [14, 15]. In the past, intervention efforts in low-income countries have been guided by simple mechanistic views of contexts and systems [16]. For example increasing supply of new guidelines and trainings although evidence from higher income settings indicates that this alone usually fails to change performance [16, 17].

The challenge therefore remains to identify implementation strategies that go beyond addressing knowledge, skills or the lack of resources, but address the issues of supporting health systems to deliver better services through implementation of evidence-based cost-effective interventions.

The CODES [18] project focuses on scaling up child survival interventions by learning how to resolve bottlenecks to access to care and determining which set of evidence-based strategies are most likely to increase coverage. The project is helping to build sustainable and scalable capacity of District Health Teams (DHTs) to effectively implement existing national strategies and works within the district planning cycle. It also places great emphasis on strengthening community demand [19]. While the various tools combined by the CODES Project have previously been used individually primarily at national level; the CODES Project seeks to adapt and combine them in an integral way for implementation at the district level.

This paper presents early experiences from the 2 years of adaptation, alignment and implementation of the tools in five districts in Uganda.

## Methods

### CODES project description

CODES is a district focused health systems management and community empowerment project that seeks to improve effective coverage and quality of child survival interventions. CODES combines UNICEF tools designed to systematize priority setting, allocation of resources and problem solving with Community dialogues based on Citizen Report Cards and U-Reports used to engage and empower communities in monitoring health service provision and to demand for quality services. The first 2 years of the project were a proof of concept, during which time the tools were adapted to the local situation and their adoption piloted in five districts. The Project has two implementing partners, Child Fund International (CFI), Liverpool School of Tropical Medicine (LSTM), who work together and are responsible for the supply side and Advocates Coalition for Development and Environment (ACODE) that is responsible for the demand side.

**Fig. 1:**
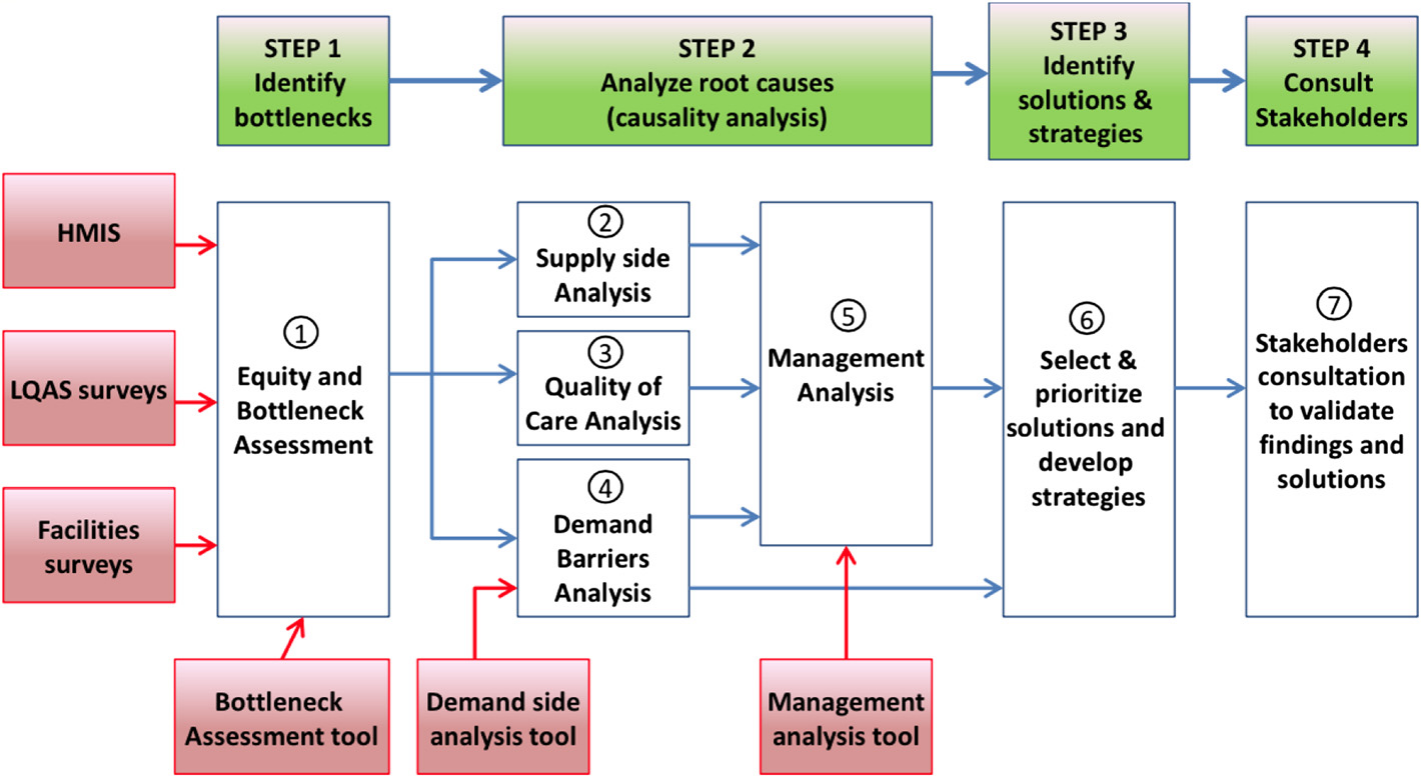
Schematic representation of CODES intervention components

The tools combined by CODES include; LQAS, Bottleneck analysis, Causal analysis, Continuous Quality Improvement (CQI), Community Dialogues based on Citizen Report Cards and U reports. Figure 1 shows how the tools were supposed to come together and reinforce each other. Lot Quality Assurance Sampling (LQAS) surveys were used to generate district specific data on service provision and healthcare use at community and health facility levels [20]. The surveys focused on availability of essential healthcare inputs; healthcare seeking behavior, coverage and quality of high impact interventions or practices at both community and health facility levels. The LQAS was also used to classify Supervision Areas as high or low performing relative to four predetermined standards set in consultation with program managers [21]. The data were then aggregated at district level and presented in the form of percentages with regard to coverage of interventions.

Based on the data generated through LQAS a bottleneck analysis tool was then used to assess health system constraints. Using the Tanahasi Model the DHMTs with support from CFI/LSTM identified bottlenecks in the operation of the health system; analyzed the constraining factors responsible for bottlenecks and selected effective measures for improving service performance and quality. Each DHMT identified five key bottlenecks affecting quality delivery of interventions for managing diarrhea, pneumonia, immunization and malaria at health facility and community (VHT) level. ACODE conducted a similar analysis of high-priority demand-side bottlenecks in one randomly selected community per Supervisory Area, for each district. All the information was then fed into the Tanahasi model which generated graphic displays that were presented to the district health teams, planners and policy makers for discussion.

For each of the selected bottlenecks an in-depth analysis was conducted to find the causes of the bottlenecks and of the management issues contributing to the bottlenecks. The causal analysis tool enabled DHMTs to determine areas needing management capacity-building and to reprioritize schedules for supervisory visits guided by the performance of the different supervision areas. Possible solutions and strategies were then identified and incorporated into the district annual health work plans with more resources being targeted to low performing Supervision Areas of the district [21].

Continuous Quality improvement (CQI) was used to aid the implementation of the identified priorities. CQI is meant to involve managers and service providers in continuous improvement of work processes to achieve better outcomes using the Plan, Do, Study, and Act (PDSA) cycles of the Quality Improvement (QI) Model approach to address priority problems at district and Health Facility (HF) levels. District and Health Facility Quality Improvement Teams were set up in all the pilot health facilities and were trained, mentored and supported by the implementing partner. Two Peer-to-peer learning [22, 23] workshops were held to facilitate DHMTs to share and learn from each other’s experiences and innovations.

In order to engage and empower communities in monitoring health service provision and to demand for quality services, Citizen Report Cards (CRCs) were developed with factual information generated from the LQAS and qualitative surveys to facilitate community dialogues. The CRCs were initially piloted and then revised based on the feedback before rollout. Each district had a Citizen Report Card that comprised of two A4 sheets presenting district specific quantitative and qualitative information on immunization coverage, utilization of services for pneumonia, diarrhea and malaria for children under five, availability of essential commodities, availability of human resources, geographical accessibility, initial, adequate and continuous utilization of health service for children under five. Communities during the Community Dialogues based their opinions, action plans and strategies on the information published in the CRCs. The CRCs also enabled the local communities to make their own appraisals, analyses, and plans and to monitor and evaluate the results. Additional file 1: Figure S1 shows a sample of a CRC.

Community dialogues were intended to mobilize and galvanize communities to demand effective and quality health service delivery and to promote accountability through the use of SMS media platforms such as U-Report [24]. Equity was a consideration in organizing Community Dialogues thus enabling underserved communities to articulate their barriers to access and utilization. The Community dialogue brought together 70 to 100 people comprised of parents/caretakers of children under 5 years, community leaders (political, cultural, religious and social), VHTs and health workers. The dialogues lasted 2 days. In order to get the participants to commit to the 2 days and to keep time breakfast and lunches were served at the venues. On the first day after the initial introductions the group was divided into four breakout sessions comprised of; caretakers, VHTs, community leaders and health workers. Health workers held their sessions at nearby health units while the other three shared a venue. During these sessions each of the breakout groups brainstormed on the problems that they face with health care utilization and delivery; discussed which of the problems required government assistance to address and which problems they could as communities address with relatively little if any assistance from outside groups or funders. As part of this process each group was required to name the problem, the cause, and the solutions, those responsible for addressing the problem and who would take responsibility and when the activity would begin. During the second day the groups were brought back together for an interface meeting between the community of users and health service providers. Together the groups identified overlapping action steps developed the previous day and action steps identified by each of the groups that required the activities and buy-in of other groups. All participants then voted on a dialogue wide contract and elected a CODES committee to oversee the activities. In order to get the communities to own the process CODES Committees were established with committee representatives from each of the villages represented to ensure follow through of the action plans developed. Post dialogue follow-up was conducted to establish the improvements that had occurred as a result of this intervention. During the dialogues the participants were also introduced to U-Report an SMS monitoring tool that was both free and anonymous. Participants were asked to register and use it as a means of providing feedback. Following the community dialogues ACODE sent out U-Report surveys that asked respondents about their general participation in any of the post-dialogue activities.

### Study setting: service delivery

Uganda’s health services are devolved to District Local Governments (DLGs) and delivered by the district health services led by the District Health Teams (DHTs) [25]. Basic health services are provided through a referral structure consisting of; Village Health Teams (comprised of community volunteers), and health facilities (including Health Centres II, III and IV and hospitals). Most districts have NGOs and other agencies operating in the health sector but most of their interventions are managed vertically and are not always in line with district priorities. While district health office has control over funds allocated by the central government for health, most of this funding is usually ear-marked, leaving the district health managers with minimal “fiscal space” for re-allocation of resources to address needs identified at district level.

In 2009, the standard Health Facility Population Ratio for health centre IV, III, and II was 1: 100,000, 1: 20,000 and 1: 5000 respectively. The Actual situation for health centre IV, III and II was 1: 187,500, 1: 84,507 and 1: 14,940 respectively [26]. Districts are catering for a substantial proportion of the population with DHTs making decisions, planning and providing services for this proportion of the population.

Uganda represents an excellent real-world opportunity to examine the effects of developing a prioritized package of interventions. Uganda has developed a comprehensive National Child Survival Strategy 2010–2015 [27], with 15 interventions at family/community level, 12 at population level, and 17 at health facility level to be delivered by village health teams.

### Study design

This was qualitative study. This design was chosen because it allows for in-depth exploration of the experiences of the DHTs and other partners in the adaptation, integration and implementation of the various tools [28, 29].

### Study sites and selection criteria

The study was conducted in five districts in Uganda, Bukomansibi, Masaka, Buikwe, Mukono and Wakiso. The criteria used by the CODES project to select the districts included: high child mortality rates and the representation of both new and old districts. Bukomansimbi and Buikwe were both new districts, while Mukono, Wakiso and Masaka were old districts.

### Study participants and sample size

Thirty eight participants were selected for the study. These included members of the DHTs, district planners, Chief Administrative Officers in the five pilot districts and officials from the two implementing partners. As this was a qualitative study the sample size was determined by data saturation point, a point at which further interviews generate no new information about the subject of investigation.

### Study team

The research team consisted of a Ugandan Social Scientist experienced in qualitative research (AK), two Ugandan public health specialists (DKH) and (PW) with previous experience as Heads of a DHT, a Ugandan research assistant (ES), a Ugandan biostatistician (DB) and a Swedish health systems specialist (SSP) with experience in health systems including Uganda. No one on the team was working within the district health system.

### Sampling method for participants

The research participants were selected using a purposive sampling method [30]. This method allowed for selection of participants based on their knowledge and participation in CODES related activities.

### Participants’ recruitment procedure

The participants were invited to participate in the study through the DHO’s office. Appointments were then arranged by the research team and face-to-face interviews conducted.

### Data collection method

The study was conducted from January 2012 to December 2013. Individual interviews (IDIs) were used to collect data. This was the method of choice because it allowed the participants to reflect on their individual experiences which would be more difficult in focus groups. All IDIs were conducted in English since all the participants were fluent in English. During the interviews the study participants were asked to reflect and describe [31] the processes they normally went through when planning and setting priorities for their districts; their experience with the CODES project, the usefulness of the tools; their willingness to continue using them and their perceived constraints. Probing was done to get a deeper understanding of the experiences [32, 33]. Audio recording of the interviews was done. Participants’ confidentiality was maintained. Each interview lasted at most 60 min.

### Interview guide

A semi-structured interview guide was used for data collection. The guide was specifically developed with the aim of understanding from the perspective of the District Health Management Team members their experiences in the adoption and alignment of the CODES intervention within the district planning processes in the five pilot districts. The guide further explored their perspectives regarding the usefulness of the CODES intervention and their willingness to adopt the various tools as part of their routine efforts in district management capacity strengthening and empowering communities to utilize and demand quality health services.

### Data analysis

Data from the audio recording device was transcribed verbatim. Data from the interviews were supplemented by observations during the implementation and by information extracted from the implementation reports and district plans [34]. Interview transcripts were read a number of times to get a general understanding of the material and the emerging themes coded. Thematic analysis [35] was used to distill the experiences of adoption and implementation of the intervention and the lessons learnt in the process.

### Ethical considerations

Ethical clearance was obtained from Uganda National Council for Science and Technology (UNCST-SS 2548) to conduct this study. Permission to conduct the study was also sought from the District Health office in all the five districts. Individual consent was obtained from all the participants prior to being interviewed.

## Results

### Improving management capacity

#### Planning and coordination

In the initial conceptual design of the project (Fig. 1) the demand and supply side tools and processes were closely interlinked and complimented each other. In reality however that was more difficult to accomplish due to the differences in time needed to adapt the supply- and demand-side tools and to synchronize the activities with the district planning cycle. The supply side tools were easier to adapt and took a shorter time, the demand side tools like the CRCs had to be developed, translated and pretested. The community dialogue process also needed to be piloted before being rolled out in the districts. Since these activities were designed to feed into the districts’ planning cycle there wasn’t much time available for joint planning. Coordination was mainly at workshop level where the implementing partners and district officials all came together and it was in these forums where the partners and district officials had the opportunity to share experiences and lessons learnt. Peer to Peer sessions were highlighted as important forums for sharing and learning.

#### Adopting bottleneck, causal and management analyses tools

Interviews with members of the DHTs who participated in the LQAS surveys and in the follow up workshops that included bottleneck, causal and management analyses expressed appreciation not only for the knowledge and skills acquired during their training and participation but also the importance of using the data generated for evidence-based planning. They explained that this was not their normal practice. Members of the DHT expressed appreciation for what they referred to as

“*a move from an adhoc to a more systematized prioritization process through Bottleneck analysis (BNA), Causal analysis (CA) and the development of action plans to address bottlenecks*.”

They also acknowledged however that they were able to accomplish these tasks as a result of the technical support from CFI/LSTM. A member of a DHT in one of the districts explained that:

“*We were supported by CFI to conduct the LQAS, the Bottleneck analysis and the Causal analysis. We participated in the hand tabulations but not in the final analysis which generated the graphic displays. So whereas we participated in these processes I cannot say that we can now conduct them on our own we would need more training and support.*” (DHT member, Masaka district)

The management checklist was also very much appreciated. Members of the DHT in one district explained that they did not normally consider management issues in their planning process:

“*This is a new way of thinking about management; it was an eye opener for us.*”

It was also noted, however, that some of the management bottlenecks that they identified had to do with the very limited fiscal space available at the district level thus making it difficult for them to address the identified bottlenecks. A member of a DHT pointed that:

“*In our district we have insufficient managerial capacity at sub-county and at the lower levels but we have very limited resources available to conduct regular support supervision so we cannot address the problem.*” (DHT member, Wakiso district)

During the second year of implementation, the, BNA, CA and MA tools’ were combined during implementation as was the original design of the intervention. Members of the DHT appreciated this more than when tools were implemented separately. A member of the DHT explained that:

“*Doing the BNA, CA and MA together helped us better understand how the different processes are related and how they feed into one another unlike in the first year when they were conducted separately.*”

Interviews with the implementing partner also revealed that by combining the three tools they saved time and resources and were able to go through all three tools in a relatively short time.

#### Work plan development–planning with available resources and consideration for equity in access

Each of the districts went through the processes of bottleneck, causal and management analyzes twice and developed district health operational work plans based on evidence from these tools. With support of the slash fund from UNICEF each of the districts during the 1st year were able to implement some of the priority activities included in their work plans. However due to financial constraints that included both limited decision and fiscal space, not all priorities identified were allocated resources. A member of the DHT in one of the districts explained that:

“*We receive two kinds of grants from central government, conditional and unconditional. The financial resources received under the unconditional grants are very limited indeed there are so many competing priorities that every year we have unfunded priorities that we carry forward to the next year hoping to get additional resources but we don’t.*” (DHT member, Buikwe district)

Two of the districts however organized partner meetings, and through these meetings were able to secure support and resources for some of their priority activities that they identified. Other districts utilized the slush fund to implement some of the prioritized interventions. For example the slush fund enabled two of the districts to conduct IMCI training for their health workers and to start monitoring childhood pneumonia, diarrhea and malaria indicators. In another district it enabled members of the DHMT to conduct more frequent support supervision and to hold meetings as illustrated by the following quote from one of the members of the DHT.

*The slush fund enabled us to conduct more frequent support supervision, before we were struggling to reach all the different units, now we have been able to cover all the different units more than once the challenge is going to be when there is no slush fund. We had also hoped to give refresher training for the health workers but we are still looking for resources.*” (DHT member, Wakiso district)

Members of district health teams expressed willingness to continue improving on the planning process and to be more systematic when prioritizing child survival interventions as articulated below:

“*…Good enough after knowing our weaknesses, what we did first was to sit, analyze our data then started planning. So this data actually assisted us a lot in planning and we had to sit with the ACAO because she is a member also and is attached to health and at the same time we sat with the planner and secretary for health.*” (DHT member, Bukomansimbi district)

“*We have used the findings from the project to lobby for more support from the district and to also approach our development partners for more support.*” (DHT member, Buikwe district)

It was noted however that conducting LQAS might be a challenge in future as one of the members of a DHT explained:

“*At district level we do not have the resources to implement LQAS. We normally use data from the HMIS but it is not as complete or as detailed as the data generated by the LQAS so we will need to see if perhaps the LQAS can be taken on by the district as a whole because we do not have those kinds of resources in our health budget.*” (DHT member, Masaka district)

Although poor-performing supervision areas were identified through LQAS, this data did not always filter through to the prioritization and planning process in order to ensure equity focused planning which is a major priority for CODES. In the district plans reviewed there was no evidence of this data having influenced the prioritization of areas to be focused on.

#### Peer-to-peer learning

Peer to peer learning was an inbuilt mechanism within CODES to promote the sharing of experiences between districts. DHT members interviewed reported that the peer to peer workshops enabled them to exchange ideas and best practices with other districts implementing CODES activities. They further explained that it enabled them to carry out a self-assessment and analyze their unique contextual features to identify what could work best in their own environment as illustrated by the following quote.

“*As we shared the experiences we got to learn from others because some others had interventions which they had already applied and we were able to learn from them. These peer to peer interventions are good because you learn from others and you get to know how they approach this then you come and try it in your district*.” (DHT member, Buikwe district)

DHT members described the peer-to-peer learning meetings as ‘*a mechanism for joint learning and reflection*,’ which was appreciated. These meetings, according to members of the DHTs interviewed, ‘*motivated them and enabled them to learn useful practices through interacting with peers in an organized forum*.’

“*First of all, we learnt a lot from other districts. We were challenged to hear that some district had gone far. Districts like Mukono have gone further and carried out the partners meetings. Basing on the lesson leant from peer to peer; we are trying also to adopt some of these …so that we can improve*” (DHT member, Masaka district)

This activity should be kept as part and parcel of the intervention in future since it promotes peer sharing and learning.

#### Continuous quality improvement

While the CQI teams in the districts expressed appreciation for the processes of CQI, the implementing partner responsible for this component, reported that motivation for CQI was an issue. The management checklist designed for managers to assess their own performance however was appreciated by the district health managers as illustrated by the following quote from a member of the district health team

“*The management checklist is helping us as leaders on how to improve our day to day work, how to manage our departments. It has helped us to improve our leadership. We identified bottle necks for which we did not have any funds but we requested some of our partners to help us for example Mildmay. We are confident this can help us improve on service delivery*.” (DHT Member, Mukono)

It is evident however that intrinsic motivation may not be enough to sustain efforts of CQI in a context of shortage of health workers and low wages. More extrinsic motivation maybe needed to sustain it.

### Community empowerment and creating demand for health services

#### Citizen’s report cards (CRC)

CRCs were developed as a basis for Community Dialogues. They portrayed district specific data generated through the LQAS surveys as well as qualitative surveys conducted. Reports from the pretest of the CRC revealed that people appreciated them especially the smiley and sad faces as well as the information that was portrayed on them about their districts. According to ACODE’s pilot report, mothers and caretaker in the rural setting who had lower levels of formal education

*.. Were less able to understand and interpret the CRC compared to those who participated in the first FGD. In the second group, the participation rate was low, even when participants were individually asked for a response. Their interface with the CRC was also low, with most viewing it as an alien cosmetic document. They suggested that the statistical data should be presented with illustrations, similar to those used to present the qualitative data findings*. (ACODE, 2012, Pretest Report)

Another criticisms had to do with the qualitative data presented in the CRCs. Five key findings from the qualitative baseline study were included on the CRC in response to the question *why do children sometimes fail to get the medical care they need?* The responses that cut across the five districts that were summarized by ACODE included the following:

- *Abusive health workers*
- *No transportation* /*health facility too far*
- *Mothers sharing doses among children or not giving full doses to children*
- *Health workers requesting illegal fees*
- *No medicine in the health facilities*

These qualitative findings however drew some criticisms from some of the DHT members who felt that some of the issues presented as cross cutting were not true in all communities. In future it might be useful to reflect further on what qualitative data is useful to present on the CRC.

#### Community dialogues

Community dialogues were facilitated by the implementing partner and were based on the CRCs. In organizing the dialogues equity was a consideration and this enabled voices from the underserved communities to articulate their barriers to access and utilization. Some of the outcomes of the community dialogues included community contracts with action points and selection of committees to continue the process. This process, to some extent, empowered communities to make decisions and to take action. ACODE reported that in Bukomansimbi District:

*After the community dialogue in February 2013, all stakeholders embarked on implementing the agreed upon actions and the district implemented activities using the slush fund. These among others included the training of VHTs and health workers in ICCM and IMCI. When ACODE next visited this facility five months after the dialogue, the facility head reported some of the improvements which he attributed to the effects of this dialogue. Some of the changes included; the increase in OPD attendance by children from 90 in January 2013 to 128 by June 2013. OPD attendance by adults had also skyrocketed from 562 to 2371 during the same period. Immunization coverage had also reported increased. Parasitological tests for children under four years increased from 45 to 90 while those of children above five years increased from 138 to 615 from January to June. The community dialogue helped the community members in realizing that the services were available in the health facility and the training that health workers received enabled them improve on treatment of cases. In all we realize increased service utilization in this area (Makerere University/Karolinska Institutet, 2013, Synthesis Report).*

The community dialogues, however, did not build on existing structures in the districts and communities. There were also concerns expressed by members of the DHT about their lack of involvement in the implementation of the community dialogues although other health providers at unit levels were involved.

#### U-report and community oversight

During the community dialogues members were encouraged to register in order to participate in U-reporting, and while many members did register the response rates were low and didn’t serve the intended oversight function. ACODE noted however that while the response rates were low the rate was:

*.. within the median response rate for U-Report’s other surveys, which suggests that there’s not something uniquely problematic about CODES’s questions or surveying methods. Additionally, given the high rate of respondents who answer “yes,” there seems to be some selection bias in favor of those who have participated in a CODES activity.*

This raises questions as to whether this might be because the audience targeted are poor, illiterate mothers who might not even have mobile phones and perhaps do not even know how to send an SMS. U-reports have been more successful among mainly young people who are more comfortable with ICT [24].

## Discussion

Findings from this study demonstrate that health systems in low income settings can adopt and implement tools to facilitate improved targeting and planning of interventions designed to improve child survival in a relatively short timeframe especially where participation across the health system is significant. The LQAS was adopted and used to collect locally relevant data to determine whether benchmarks have been reached and to aggregate the data in order to look at broader progress in the study areas [36]. Then based on this data the bottleneck analysis tool enabled the identification of major bottlenecks on supply as well as demand side for increased coverage of priority interventions [37]. The adoption of CQI principles allowed the CQI committees formed at district level to tackle some of the identified district management bottlenecks in order to close performance gaps in incremental, smaller problem-solving steps [38, 39]. Combining the supply and demand side tools however become difficult due to the differences in time needed to adapt the different tools and the time needed to synchronize the activities with the district planning cycle. So coordination between the demand- and supply side activities was not always easy and needs to be worked on in future.

Although CFI/LSTM worked closely with members of the DHT in each of the five districts, they took the lead in the implementation of the tools at district level during both the first and second year of implementation. This was justifiable since the tools were new to the DHTs and they needed to learn how to use them and feel confident to implement them. The time available for the DHTs to consolidate the skills needed to independently implement the different tools however was insufficient. This suggests that more time needed to be made available for the DHTs to consolidate their knowledge and skills to implement the tools and a strategy developed for the implementing partner to gradually withdraw as the DHTs become more confident with the tools implementation.

The importance of community empowerment through community dialogues based on CRCs cannot be understated as it created an important platform through which the community and service providers were able to have constructive dialogue. Community dialogues based on CRCs and U-Reports did promote community involvement in monitoring health providers and community-based activities with the potential of improving care-seeking and social accountability from service providers [40]. The demand side implementing partner however took the lead in the implementation of nearly all the demand side tools with very minimal involvement of district level partners. This was justifiable in view of the fact that the tools first had to be developed and then piloted before handing them over to district level partners to implement. The early identification and engagement of district level structures however could facilitate faster adoption of the demand side tools at district level.

Nearly all the districts had partners collaborating with the district health office on different health programs. Their early involvement in this process would be helpful especially in the implementation of the identified priorities. This was evident in the districts which organized meetings with their partners to discuss how some of the identified priorities could be funded.

Tools such as text message surveys have proven powerful in improving health system accountability, as have citizen report cards. The low U-report response however raises questions as to whether this is the best method given the target audience of care givers that may not be literate and have limited ability to use mobile phones. More research is needed to establish a better mechanism of getting feedback from the communities.

### Study limitations

This being a qualitative study, it did not allow for a large sample size and therefore findings cannot easily be generalizable. The IDIs also have a risk of response bias which is a tendency of some respondents to tailor or distort their responses and the most common to provide a favorable picture of the subject of investigation [41]. Even with these limitations the results and conclusions can be interpreted in the context of the district health system in a decentralized governance system like Uganda and in other low resource setting.

## Conclusion

The CODES project represents a combination of tools that attempt to deal with many of the gaps that have been identified to the successful implementation of strategies to reduce pneumonia and diarrhea deaths at district level. It focuses—on priorities, on specific bottlenecks to implementation, on local managerial gaps and on evidence-based solutions. It is important therefore to devise ways to improve the joint conceptualization of the implementation of the demand- and supply side tools. These could be though joint planning and review meetings.

Other partners for health in the districts are vital in the implementation of identified priorities. It is therefore important to involve these partners in the early stages of implementation as was mentioned by some district members in the Peer-to-peer workshops. The peer-to-peer learning sessions ought to be maintained and timed early in the planning cycle to enable lessons learnt to be integrated into the district health work plans. The Quality Improvement processes should, be embedded in ongoing activities at all levels in the district. Given that CRCs are critical in the community dialogue process they should in as much as possible be based on data that most community members can relate to. To ensure sustainability of the Community Dialogue model, it should be aligned with already existing structures: e.g., Barazas (Barazas are a Presidential initiative that was adopted in 2009 to create a platform for the citizens of Uganda to participate in the development cycle through effective monitoring and demand for accountability of the use of public resources in the delivery of services at local Government Level) [37], Village Health Teams (VHTs) and health unit management committee (HUMCs), parish development committees that were not adequately engaged.

Findings from the study demonstrate that tools targeting improving district management capacity and community empowerment can be adopted and used at district level with decentralized systems like Uganda. This was clearly articulated by DHTs who perceived the CODES project as an intervention whose systematic nature could improve prioritization of interventions that can improve child survival and are context specific.

## Additional file

Additional file 1: Figure S1. Citizen Report Card. (PDF 378 kb)
