# Supplementary material for: Improving child survival through a district management strengthening and community empowerment intervention: early implementation experiences from Uganda
Source: BMC Public Health. 2015 Aug 19;15:797. doi: 10.1186/s12889-015-2129-z (PMC4545317; doi:10.1186/s12889-015-2129-z)
Supplement: Additional file 1: Figure S1. — Citizen Report Card. (PDF 378 kb) [file 12889_2015_2129_MOESM1_ESM.pdf]

CITIZEN REPORT CARD

BUKOMANSIMBI DISTRICT

Sub-county: [All the sub-counties within a Supervision Area]

Households with Small Children

Indicators	Score (out of 100%)		Remarks
	Sub-County	District	
COMPLETE CARE: Immunizations			
Children 12–23 months fully immunized before their first birthday		41%	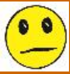
COMPLETE CARE: Pneumonia, diarrhoea, and malaria			
Children under age 5 with cough and difficulty breathing who were treated with nationally recommended antibiotics within 24 hours of the child becoming sick		65%	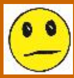
Children under age 5 with diarrhoea who were treated with nationally recommended medicine (ORS and zinc supplements) within 24 hours of the child becoming sick		9%	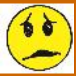
Children under age 5 with malaria who received the nationally recommended treatment (ACT) from a health facility or VHT within 24 hours of the of the child becoming sick, and who took the full dose for the required period		9%	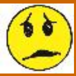
Mothers of children under age 5 who reported that their children received a malaria test when they had a fever		7%	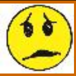
INITIAL USE OF SERVICES: Seeking help for pneumonia, diarrhoea, and malaria			
Children under age 5 with cough and difficulty breathing whose mothers sought treatment within 24 hours of the child becoming sick from a:<br>Health facility<br>VHT		 19%<br>16%	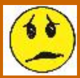
Children under age 5 with diarrhoea whose mothers sought treatment within 24 hours of the child becoming sick from a:<br>Health facility<br>VHT		 20%<br>21%	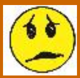
Children under age 5 with a fever whose mothers sought treatment within 24 hours of the child becoming sick from a:<br>Health facility<br>VHT		 21%<br>34%	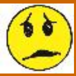

Health Facilities

Indicators	Score (out of 100%)		Remarks
	Sub-county	District	
MEDICINES: Diarrhoea, pneumonia, and malaria			
Health facilities that had no stock-outs of ORS for diarrhoea		64%	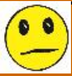
Health facilities that had no stock-outs of zinc for diarrhoea that lasted more than 1 week		29%	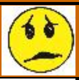
Health facilities that had no stock-outs of amoxicillin for non-severe pneumonia that lasted more than 1 week		43%	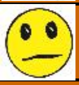
Health facilities that had no stock-outs of ACTs for malaria that lasted more than 1 week		86%	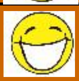
HUMAN RESOURCES			
Health facilities that have the nationally recommended number of nurses, clinicians, and doctors attending to children under age 5		7%	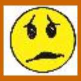
Health facilities whose nurses, midwives, and doctors were paid in the last month		100%	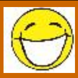
Health facilities in which health workers were trained in the last year to treat malaria, diarrhoea, or pneumonia in children under age 5 (or were trained in Integrated Management for Childhood Illness) in the last year		14%	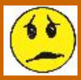
Mothers of children under age 5 who reported having to pay any money in order to have their children examined at a public health facility for diarrhoea, pneumonia, or malaria		36%	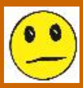
Health facilities that received external supervision to make sure that they are following national guidelines for both pneumonia and diarrhoea treatment for children under age 5		71%	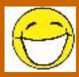
Health facilities that follow the nationally recommended guidelines for treating malaria		64%	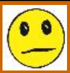
Health facilities that follow the nationally recommended guidelines for treating pneumonia		7%	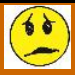
Health facilities that follow the nationally recommended guidelines for treating diarrhoea with blood		0%	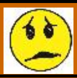
INFRASTRUCTURE			
Health facilities with emergency transport (such as an ambulance) for the care of children under age 5 on the day of the survey		21%	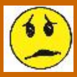
Health facilities that display child-health user fees where children get treated in the facility		14%	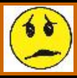

Village Health Teams (VHTs)

Indicators	Score (out of 100%)		Remarks
	Sub-county	District	
MEDICINES: Diarrhoea, pneumonia, and malaria			
VHTs that had no stock-out of ORS for diarrhoea that lasted more than 1 week		95%	
VHTs that had no stock-out of zinc for diarrhoea that lasted more than 1 week		88%	
VHTs that had no stock-out of amoxicillin for non-severe pneumonia that lasted more than 1 week		18%	
VHTs that had no stock-out of ACTs for malaria that lasted more than 1 week		22%	
HUMAN RESOURCES: Knowledge and community satisfaction			
VHTs who know the recommended treatment for malaria		100%	
VHTs who know the recommended treatment for diarrhoea		99%	
VHTs who know the recommended treatment for pneumonia		100%	
User satisfaction with the VHT services offered (those who are very satisfied or fairly satisfied)		84.6%	
TRAINING: Refreshers, supervision, and reporting			
VHTs who received basic VHT training		100%	
VHTs who received a refresher training in community-level case management (ICCM) in the past year		34%	
VHTs that were supervised by health facility personnel in the community		55%	
VHTs that kept updated records of their work in the community		18%/ 16%	

A Note on the Data: All findings represented in this scorecard are the result of a baseline survey for the CODES project that was undertaken in late 2011 and early 2012 by Child Fund International, the Liverpool School of Tropical Medicine, and Advocates Coalition for Development and Environment. All data on diarrhoea, pneumonia, and malaria in children under age 5 reflect the rates of incidence within two weeks prior to data collection. All data on health facility and VHT stock-outs reflect the rates of incidence within three months prior to data collection. The term "stock-out" refers to a situation in which no drugs are available for distribution.

Why children sometimes fail to get the medical care they need

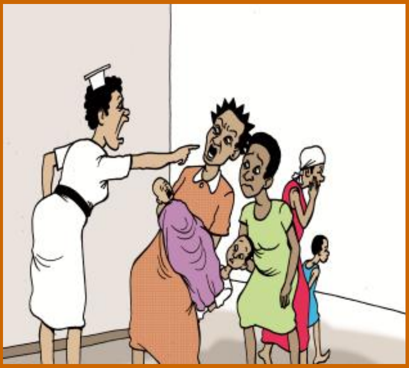

Abusive health workers

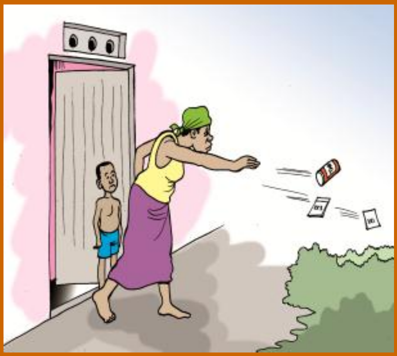

Mothers not giving full dose to children

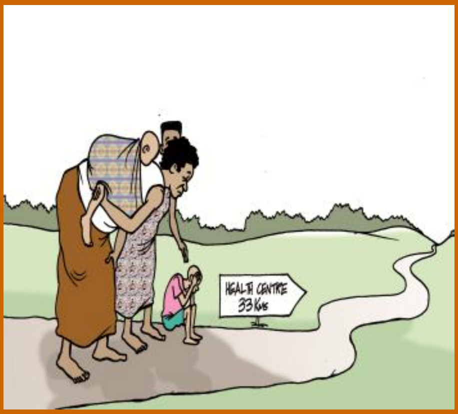

No transportation / health facility too far

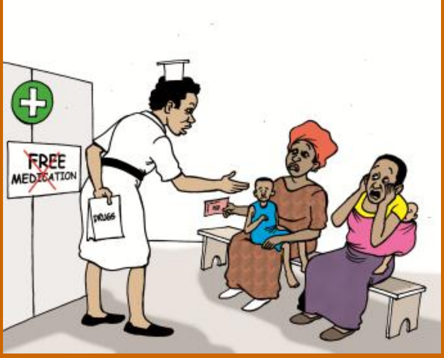

Health workers requesting illegal fees

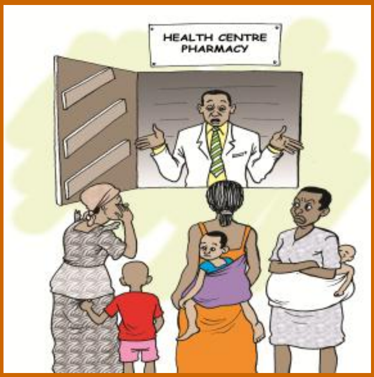

No medicines in health facilities

This Citizen Report Card was created by the Advocates Coalition for Development and Environment in Kampala, Uganda, with funding from UNICEF and the Bill and Melinda Gates Foundation, and with technical input from Child Fund International and the Liverpool School of Tropical Medicine.

Community and District Empowerment for Scale-up (CODES) is a five-year project funded by the Bill and Melinda Gates Foundation and United Nations Children's Fund (UNICEF) aimed at ensure that the Government of Uganda attains the capacity—particularly at the district level—to implement public policies and interventions that lead to quantitative and qualitative improvements in health outcomes, especially the control of malaria, pneumonia, diarrhoea in children. The project has two phases, the first of which is two years and involves five districts: Buikwe, Bukomansimbi, Masaka, Mukono, and Wakiso. CODES is spearheaded by the Ministry of Health in partnership with District Local Governments, the Advocates Coalition for Development and Environment (ACODE), and Child Fund International (CFI). Makerere University School of Public Health (MUSPH), the Liverpool School of Tropical Medicine (LSTM), and Karolinska Institutet (KI) provide technical assistance.
